# Liver metastasis and resistance to immunotherapy in microsatellite stable colorectal cancer. A literature review

**DOI:** 10.3332/ecancer.2024.1771

**Published:** 2024-09-18

**Authors:** Marcelo Porfirio Sunagua Aruquipa, Mauro S Donadio, Renata D Peixoto

**Affiliations:** 1 Gastrointestinal Oncology Department, Oncoclinicas, São Paulo 04513-100, Brazil; 2 BC Cancer Agency, Vancouver, BC V5Z 4E6, Canada; a https://orcid.org/0009-0009-0207-0022; b https://orcid.org/0000-0002-4705-4802; c https://orcid.org/0000-0003-0053-7951

**Keywords:** colorectal cancer, liver metastasis, microsatellite stable, immunotherapy

## Abstract

**Background:**

Microsatellite stable (MSS) metastatic colorectal cancer (CRC) remains predominantly managed with chemotherapy. The use of immunotherapy, whether alone or in combination with other systemic or local treatments, displays limited success, especially in the context of active liver metastases (LM). The mechanisms responsible for this resistance are not fully understood.

**Methods:**

We conducted a comprehensive search across electronic databases such as Medline, PubMed, Google Scholar and ScienceDirect. This search targeted translational studies evaluating the liver tumour immune microenvironment and immune tolerance mechanisms in CRC with LM and prospective studies that assessed immunotherapy either as a standalone treatment or in combination with other systemic or local therapies for patients diagnosed with MSS CRC. Our primary objectives included elucidating the mechanisms of resistance originating from LM in a non-systematic literature review and presenting a summary of the outcomes observed in prospective trials utilising immune checkpoint inhibitors (ICIs), with a focus on the presence of LM.

**Findings:**

There were 16 prospective trials evaluating immunotherapy for metastatic CRC comprising 1,713 patients. Response rates to immunotherapy inpatients with colorectal liver metastases (CRLM) varied from 0% to 23%. Overall, reduced or null responses to immunotherapy in the presence of liver metastasis in comparison to patients without liver involvement were observed.

**Conclusion:**

Studies consistently show the resistance derived from classical ICI, both alone and in combination with other systemic treatments in patients with CRLM. The design of upcoming trials using immunotherapy should consider LM as a stratification factor or contemplate excluding patients with liver involvement.

## Introduction 

Colorectal cancer (CRC) stands as the third most prevalent malignancy globally in both genders and ranks second in cancer-related mortality based on Global Cancer Observatory data [1]. Approximately 20% of CRC patients exhibit distant metastasis upon diagnosis, while nearly half of the individuals initially diagnosed with localised CRC eventually progress to metastatic disease [2]. Due to the unique microenvironment of the liver and the predominance of the hepatic portal venous system in collecting intestinal blood, the liver becomes the primary site for CRC metastasis, affecting up to 50% of patients with colorectal liver metastases (CRLM) during the disease course [3–5]. Regrettably, less than a third of CRLM patients qualify for curative-intent metastasectomy, leading to a significant portion succumbing to liver failure [2–4].

Mismatch repair deficient (dMMR)/microsatellite instability-high (MSI-H) status is detected in roughly 7%–10% of all CRC cases and only in 3%–4% of those with metastatic disease [6]. Dysfunction in MSH2, MLH1 and MSH6 gene products hinders the identification of mismatched and unpaired bases, resulting in an abundance of abnormal proteins (neoantigens) that are more prone to immune system recognition, particularly by tumour-infiltrating lymphocytes (TILs) [7]. Consequently, this minority subset of patients with dMMR/MSI-H metastatic CRC presents immunologically active tumours and reaps the greatest benefits from immune checkpoint inhibitors (ICIs), a recent milestone in Oncology [8].

Unfortunately, the majority of metastatic CRC cases belong to the mismatch repair proficient/microsatellite stable (MSS) subtype, where the current efficacy of ICI remains modest at best [8]. Their tumour microenvironment (TME) differs significantly from MSI-H CRC, characterised by fewer TIL and a higher presence of tumour-associated macrophages, associated with an ‘immune-excluded’ scenario [9]. Additional molecular mechanisms contributing to ICI resistance in MSS CRC encompass more frequent mutations in the antigen-presenting cell* (APC)* protein, impairing β-catenin activity [10] and heightened activation of the transforming growth factor-β (TGF-β) pathway, augmenting regulatory T cells (Tregs) within the TME while diminishing natural killer (NK) cell activity [11].

Recent efforts in research have concentrated on transforming ‘cold’ neoplasms into ‘hot’ ones, yet these endeavours have been hindered by the adverse effects of CRLM. Our manuscript aims to elucidate the immunosuppressive role played by the liver in a non-systematic literature review, delineating the contrasting outcomes observed with ICI in patients with and without CRLM and highlighting potential therapeutic strategies to surmount immunotherapy resistance in the presence of hepatic involvement. As chemotherapy demonstrates efficacy against liver metastases (LM), investigations concerning the combination of chemotherapy and ICI are not within the scope of this review article. Furthermore, data pertaining to CRC with targetable molecular alterations is not included either.

## Methodology

### Eligibility criteria

The inclusion criteria encompassed prospective studies with patients having metastatic MSS CRC published in peer-reviewed journals between 2005 and 2023, written in English, and providing essential information such as sample size, the number of CRC cases and overall response rate (ORR) according to RECIST 1.1 criteria. The presence of LM was mandatory, while other metastatic sites were allowed. The treatment regimen needed to involve an ICI, either alone, in combination with systemic treatments or liver-directed local therapies. Phase I and II studies were considered. In the same way, original research papers in preclinical and translational studies focusing on the tumoural and immune microenvironment of CRLM and their correlation with immunotherapy, were included.

Exclusion criteria encompassed articles not in English, studies involving MSI, dMMR or early stage CRC patients, duplicated papers and studies with pending results.

### Search strategy

The Preferred Reporting Items for Systematic Reviews and Meta-Analyses guidelines were used in this paper. The authors conducted an electronic search to retrieve studies. The search terms were: ‘Colon cancer’, ‘CRC’, ‘LM’, ‘MSS’, ‘Immune Checkpoint’ and ‘Immunotherapy’. The Boolean operators ‘OR’ and ‘AND’ were used in combinations with the keywords. The search for publications was conducted using the Cochrane Central Register of Controlled Trials, PubMed, EMBASE, Medline and ScienceDirect databases. The Zotero 6.0 software was used to manage and exclude repeated references.

### Selection process

The initial screening process involved the evaluation of articles based on their titles and abstracts, with the authors applying specific inclusion and exclusion criteria and removing duplicate sources. Selected papers then underwent a rigorous full-text review using the same criteria. Subsequently, a total of 70 articles were deemed suitable for inclusion in the manuscript, comprising 16 prospective trials and 54 translational or preclinical studies.

### Data extraction

In the case of prospective studies, our analysis involved a thorough examination of the full text of selected articles. We extracted key data points, including details about the authorship, year of publication, methodology employed, type of intervention, the number of patients with MSS CRC included in each study and the outcomes of interest, which primarily encompassed the objective response rate and/or progression-free survival (PFS).

### Risk of bias and quality assessment

The quality and methodology of the trials was assessed by the Cochrane Risk of Bias Tool. The domains rated were: (D1) Randomization, (D2) Deviations of initial protocol, (D3) Missing outcome data, (D4) Measurement of the outcomes and (D5) Reporting of the population of interest in trials with various tumour types, in our case MSS CRC was the population of interest. The quality of the evidence was in accordance with the grading of recommendations assessment, development and evaluation scale.

## Literature review for liver tumour immune microenvironment and immune tolerance mechanisms

### Liver tumour immune microenvironment

In the liver parenchyma, alongside the conventional hepatocytes, a diverse array of cell types coexists, each playing distinctive roles in the regulation of the liver microenvironment. Notable among these are hepatic stellate cells (HSCs), vascular smooth muscle cells, Kupffer cells (KCs), T cells, B cells and endothelial vascular cells [12]. The liver, being a unique organ, has evolved immune tolerance mechanisms owing to its exposure to the bloodstream from the gut and interaction with numerous external antigens from nutrients and commensal bacteria [13]. This characteristic immune tolerance extends to liver allografts or hetero-transplantation, which comparatively need less immunosuppressive treatment compared to kidney or heart transplants [14, 15].

The innate immune system within the liver primarily comprises KC, characterised by their expression of CD5L and CD163, functioning as mononuclear phagocytes [13]. Conversely, the adaptive immune system encompasses cytotoxic T cells expressing CD8 and CCL5, which exhibit pro-inflammatory antitumour functions, and Tregs expressing CD25, CTLA4 and FOXP3, contributing to anti-inflammatory processes [16].

Under normal physiological conditions, Treg cells are tasked with preventing autoimmunity events by downregulating cytokines, particularly IL-2. However, in the context of the TME, Treg cells assume a predominant role, forming an immune-suppressive subset that fosters tumour growth [17, 18].

### Immune tolerance mechanisms of the liver

#### Primary resistance

The primary resistance exhibited by liver metastasis cells from CRC remains a partially elucidated phenomenon. Translational studies derived from clinical trials involving ICI have shed light on certain factors influencing this resistance. Specific mutations, such as those in *b2-microglobulin* and *JAK 1/2* genes, have been identified as contributors to resistance against programmed death-ligand 1 (PDL1) blockade. Notably, this resistance seems independent of MSI

status. This resistance mechanism may be attributed to alterations in the interferon-gamma pathway, leading to errors in the antigen-presentation process to lymphocytes [19, 20].

Beyond genetic factors, tumoural cells employ evasion strategies against the innate immune system, particularly NK cells. This evasion is accomplished through the acidification of the TME facilitated by lactate production. This acidification induces mitochondrial stress in NK cells, ultimately culminating in apoptosis [21].

#### Acquired resistance (liver immune microenvironment)

**Figure 1.**
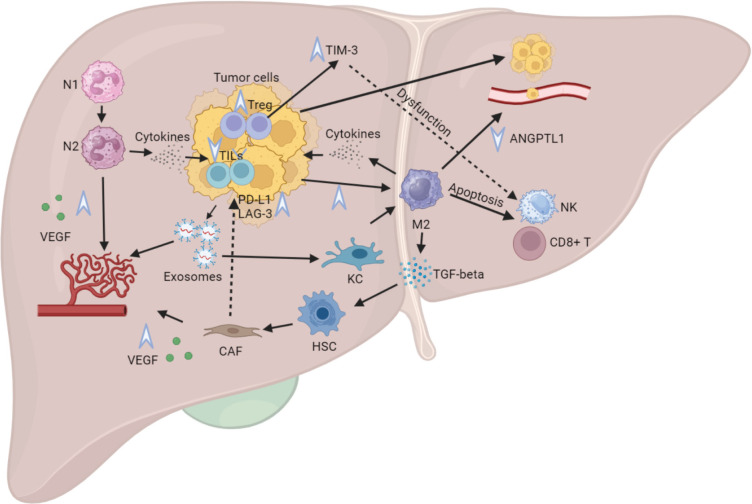
Impacts of CRLM on the hepatic immune microenvironment. Legend: Tumoural cells release exosomes that facilitate angiogenesis and enhance endothelial permeability. These exosomes also interact with KCs, prompting a shift towards a proinflammatory M2 phenotype and inducing apoptosis in NK cells and cytotoxic CD8+ T cells. The M2 macrophages, in turn, release TGF-beta, contributing to the differentiation of physiological HSCs into CAFs. Additionally, M2 macrophages downregulate ANGPTL1, resulting in heightened vascular permeability. Both N1 and N2 neutrophils stimulate VEGF production, thereby increasing angiogenesis. Furthermore, N2 and M2 cells secrete cytokines with immunosuppressive properties. These intricate interactions collectively contribute to a decline in CD8+ T cells and an elevation in Tregs, culminating in an augmented expression of TIM-3. Activation of TIM-3 induces dysfunction in NK cells and facilitates the transition of macrophages to the M2 phenotype, thereby perpetuating tumour progression and immunosuppression. N1, Neutrophil type 1; N2, Neutrophil type 2; KC, Kupffer cell; M1, Macrophage type 1; M2, Macrophage type 2; TGF-beta, Transforming growing factor beta; VEGF, Vascular endothelial growing factor; HSC, Hepatic stellate cell; CAF, Cancer-associated fibroblast; NK, Natural killer cell; TIM-3, T-cell immunoglobulin mucin 3.

Upon the infiltration of tumoural cells into the liver parenchyma, a cascade of interactions unfolds, orchestrated by various cellular components and signaling pathways. Tumoural cells initiate the secretion of exosomes, serving as mediators for angiogenesis promotion and increased endothelial permeability. This facilitates the recruitment of additional tumoural cells and immune cells within the liver [22]. These exosomes also engage with local liver macrophages or KC, inducing a shift towards a proinflammatory Macrophage type 2 (M2) phenotype (Figure 1), as described elsewhere [23].

Studies have demonstrated that metastatic tumoural cells in the liver disrupt the balance of TIL populations. This disruption involves a decrease in Macrophage type 1 (M1) macrophages with anti-tumoural effects and an increase in M2 macrophages, known for promoting apoptosis of NK cells and cytotoxic CD8+ T cells. This not only diminishes the local response in the liver but also hampers systemic T cell infiltration [24]. The M2 macrophages exhibit a pro-tumour activity by secreting TGF-β, contributing to the differentiation of physiologic HSC into cancer-associated fibroblasts (CAFs). These CAFs, in turn, interact with various pathways in the TME, fostering immune depletion [25]. Additionally, M2 macrophages downregulate the angiopoietin-like protein 1 (ANGPTL1), leading to increased vascular permeability in sinusoidal vessels for the arrival of more tumoural cells (Figure 1) [26].

An additional population of interest pertains to neutrophils, exhibiting both anti-tumour neutrophil type 1 (N1) and pro-tumour neutrophil type 2 (N2) characteristics. These cells create mesh-like structures within sinusoidal vessels lacking cytotoxic abilities. This phenomenon amplifies the attachment of circulating tumour cells within hepatic sinuses and collaborates with M2 macrophages to stimulate the production of angiogenic factors, such as vascular endothelial growth factor (VEGF) [27]. Furthermore, N2 neutrophils engage with M2 macrophages to secrete cytokines with immunosuppressive properties, notably TGF-β, thereby diminishing the density of TILs within the liver and other metastatic locales (Figure 1) [28].

Sub-analyses of trials employing immunotherapy combinations (anti-PDL1 + anti-CTL4 or anti-PDL1 + targeted therapy) in patients with MSS metastatic CRC suggest that the density of TILs could serve as a more reliable prognostic biomarker for treatment efficacy compared to tumour mutational burden [29–32]. A consistent trend is observed in other metastatic CRC trials involving immunotherapy, where the presence of liver metastasis diminishes the systemic ORR in comparison to patients without liver metastasis [33–35]. This reduction in efficacy is attributed to the decrease in TIL density, especially cytotoxic CD8+ T cells, in both primary tumours and extrahepatic sites of disease (Figure 1) [36].

These complex interplays significantly contribute to the expansion of a distinct subset of CD4+ T cells, known as Tregs, within the TME. This expansion leads to the heightened expression of the T-cell immunoglobulin mucin-3 (TIM-3) protein by Treg cells, widely regarded as a crucial modulator of immune exhaustion evident in both localised and metastatic CRC [37]. TIM-3 activation induces a dysfunction among NK cells and fosters the transition of M1 macrophages toward the M2 phenotype, establishing a cyclical process that perpetuates a milieu favouring tumour progression and immunosuppression specifically within LM (Figure 1) [38].

The planar cell polarity pathway activated by Wnt morphogens, independent of beta-catenin, such as WNT5A binding to the FZD receptor, further contributes to cancer cell proliferation. These proteins are expressed by immune cells and CAF, triggering the transcription of genes related to cell adhesion and migration [39].

Another mechanism impeding the effectiveness of immunotherapy involves the interaction between hepatocyte growth factor and the MET receptor kinase pathway. This pathway is expressed in tumour-associated macrophages and CAF, leading to the upregulation of PDL1 expression in tumoural cells along with an increase in Lymphocyte-activation gene 3 (LAG3), conferring resistance to anti-PDL1-based therapies [40].

Particularly in female patients, estrogen levels have been implicated in promoting myeloid-derived suppressor cells (MDSCs) and Treg cells, resulting in increased expression of the immunosuppressive protein TGF-b [41]. Furthermore, estrogen in the TME appears to augment the population of immunosuppressive M2 macrophages while reducing pro-inflammatory M1 macrophages, creating a challenging environment for NK cell recruitment and promoting tumour growth [42].

## Literature review on the efficacy of ICI in MSS patients with and without LM

### Anti–PD-1/PD-L1 antibodies alone or in combination with anti-CTLA-4 antibodies

The preliminary studies employing either pembrolizumab or nivolumab in patients diagnosed with refractory MSS CRC did not report any discernible treatment responses. Moreover, the results did not delineate specific outcomes concerning the presence or absence of LM in these patients [8, 43, 44]. The studies reviewed in this manuscript are summarised in Table 1.

A few clinical trials explored the combination of an anti-programmed death 1 (PD1) or PD-L1 with an anti-CTLA-4 monoclonal antibody in MSS CRC, yielding discouraging results. The Checkmate-142, a non-randomised phase II study, reported a median PFS of only 1.4 months among patients with MSS CRC (*n* = 28) [45]. Similarly, the CO.26 randomised phase II trial showcased poor median PFS for both investigational (durvalumab plus tremelimumab) and placebo arms (1.8 versus 1.9 months, respectively), with 98% of participants exhibiting MSS tumours and 70.6% harboring liver metastasis [46]. In a *post hoc* analysis of the CO.26 study stratified by the presence or absence of CRLM, individuals lacking liver metastasis who were administered durvalumab and tremelimumab exhibited enhanced PFS at 1.8 versus 2.0 months, and OS at 5.3 versus 9.4 months. Moreover, the disease control rate (DCR) notably favoured those without liver involvement, reaching 49% as opposed to 14% [47].

In a phase 1 trial involving extensively treated MSS CRC patients who had undergone three or more lines of treatment, a novel anti-LAG3 molecule named favezelimab was administered in combination with pembrolizumab. The observed ORR was reported as 0% in the favezelimab arm, while the combination arm exhibited a modest ORR of 6.3%. The median PFS was recorded at 2 months. Upon stratification based on the expression of PD-L1, patients with a combined positive score (CPS) greater than 1 demonstrated a trend towards improved overall survival with a median duration of 12.7 months, in contrast to CPS-negative patients who exhibited a median OS of 6.7 months [48].

Recent investigations into the combination of the second-generation CTLA-4 inhibitor botensilimab and the PD-1 inhibitor balstilimab demonstrated promising results in various solid tumours, including MSS CRC. Botensilimab, an Fc-enhanced next-generation anti-CTLA-4 IgG1 antibody, exhibits a higher affinity to FcγRs, potentially augmenting its anti-tumour activity while mitigating complement-mediated toxicity [49]. Moreover, this novel anti-CTLA-4 antibody enhances T cell priming, strengthens APC/T cell interactions, and activates macrophages and NK cells [50].

In a cohort of heavily pretreated MSS CRC patients from a multicohort first-in-human phase 1a/1b trial (including 25% who had previously failed immunotherapy), the combination of botensilimab and balstilimab yielded unprecedented outcomes. Notably, patients without active LM – defined as those without a history of LM or with treated/ablated metastases without recurrence – achieved a median OS of 20.9 months. Conversely, individuals with active LM had a median OS of 8.7 months. Among participants without active liver involvement, an ORR of 23% and a DCR of 80% were observed, accompanied by a manageable safety profile. However, in the presence of active LM, the ORR was 0% [51]. Encouraged by these findings, a randomised phase 2 trial in MSS CRC patients without active LM has recently completed accrual (NCT05608044).

### ICI in combination with other drugs

The preclinical evidence highlighting the potential synergy between mitogen-activated protein kinase pathway inhibition via MEK inhibitors and the heightened anti-tumour activity of PD-1 inhibitors due to increased MHC-1 and PD-L1 expression has sparked interest in their combined use [52]. Initial optimism emerged from a phase Ib trial combining atezolizumab and cobimetinib in various solid tumours, reporting an ORR of 8% in patients with metastatic CRC, predominantly exhibiting MSS tumours [53]. However, subsequent exploration in the phase III IMblaze-370 trial failed to confirm this potential synergy. Despite initial promise, no significant differences in median OS and PFS were observed among the three arms – regorafenib (control arm), atezolizumab alone and atezolizumab with cobimetinib – where 92% of participants had known MSS CRC [54].

Multitarget tyrosine kinase inhibitors (TKIs) in conjunction with ICI have shown variable outcomes. In the Japanese REGONIVO trial, combining regorafenib and nivolumab demonstrated an impressive 33% ORR and a median PFS of 7.9 months in the MSS CRC cohort, particularly notable among those without LM [32]. However, a subsequent phase II study with the same agents presented a stark contrast, showing an ORR of 7% and a median PFS of 1.8 months among metastatic MSS CRC. This study also underscored the inferior outcomes associated with the presence of LM, indicating 0% ORR in individuals with LM compared to 22% in those without [55].

Further investigations into regorafenib in combination with an ICI, avelumab in the French REGOMUNE trial, displayed a median PFS of 3.6 months and an overall DCR of 10.8 months among refractory MSS CRC; however, outcomes specific to LM were not detailed [35]. Similarly, pembrolizumab addition to regorafenib in a phase I/II trial did not yield notable responses in pretreated metastatic CRC, showing a median PFS of 2.0 months and OS of 10.9 months, although a *post hoc* subgroup analysis suggested longer PFS in patients without liver involvement and those with prior radiotherapy [56].

Explorations into other TKIs, such as cabozantinib and lenvatinib, combined with ICI for refractory MSS CRC, have demonstrated promising ORRs (23.5% and 22%, respectively) but have not explicitly delineated outcomes based on the presence or absence of liver metastasis in the phase Ib CAMILLA and phase II LEAP-005 trials [57, 58]. Recently, the conclusive findings from the phase III LEAP-017 trial were disclosed. This study compared the efficacy of a combination therapy involving lenvatinib and pembrolizumab against regorafenib or trifluridine-tipiracil for individuals with refractory metastatic MSS CRC. Notably, 70% of participants exhibited CRLM at baseline. The median OS in the investigational arm was 9.8 months, while it stood at 9.3 months for those receiving the standard-of-care regimen (HR 0.83; *p* = 0.0379), failing to reach the predetermined statistical threshold for superiority. Moreover, although the ORR was higher in the combination treatment arm, it remained modest at 10.4% compared to 1.7% in the control arm. Subgroup analysis indicated more substantial responses to lenvatinib plus pembrolizumab, particularly in populations with a PD-L1 CPS ≥1, individuals aged over 65, those with an Eastern Cooperative Oncology Group performance status of 0, the presence of RAS mutations, and notably, in those lacking CRLM, who achieved an ORR of 28.1% [59].

### Locoregional approaches in combination with ICI

It has been investigated the utility of liver-directed therapies to overcome the immunotherapeutic resistance. The investigations conducted on preclinical models have elucidated a systemic decline in antigen-specific T cell response modulated by the actions of CD11b+ myeloid cells, also called MDSC. Following irradiation, a decline in the MDSC was observed alongside an enhanced cytotoxic CD8+ T cell infiltration into the liver [24, 60]. Previous research has indicated the suppressive role of MDSCs in specific immunity [61, 62]. Moreover, a preclinical analysis revealed that MDSC could be related to diminished levels of expression of CTLA-4, PD-1, inducible T-cell costimulator (ICOS) and Ki67 in the CD8+ T cells of the liver, contrasting with heightened expression of CTLA-4, ICOS and PD-L1 in Treg cells [63]. Combining immunotherapy with locoregional liver-targeted interventions holds significant promise but necessitates further prospective clinical validation.

Studies exploring alternative protocols combining radiotherapy and immunotherapy, although offering limited clinical benefits, observed noteworthy immunological changes. Analysis revealed treatment-associated alterations in the T cell repertoire, increased T cell infiltration into the irradiated area and reversal of M2 macrophage polarisation in the TME [64, 65].

A retrospective analysis exploring immunotherapy effects in patients with MSS CRC, unresponsive to two prior chemotherapy regimens, revealed optimal outcomes in individuals without a history of CRLM. Intriguingly, patients receiving immunotherapy post-surgical removal of LM exhibited superior response rates compared to those with ongoing LM (median PFS duration: 3.0 versus 1.5 months; HR 2.37; 95% CI 1.45–3.86; *p*  <  0.001). Unfortunately, the interval between resection and immunotherapy remains unspecified. In multivariate analysis, the presence of LM during treatment emerged as the most influential factor affecting PFS during immunotherapy (HR 7.0 95% CI, 3.18–15.42; *p*  <  0.01) [36]. This study thus suggests that addressing LM before initiating ICI potentially yields a measurable clinical response to immunotherapy.

An open-label phase II investigation of radiotherapy combined with ICI supported this proposition. Metastatic MSS CRC received radiotherapy (total of 24 Gy) following two cycles of nivolumab and ipilimumab combination. Pre-radiation, 2.5% exhibited stable disease; post-radiation, stable disease rose to 25% and ORR to 10% [66]. Once again, targeting LM, in this case with radiation, augmented immunotherapy’s effectiveness.

Regrettably, these intriguing findings were not reproduced using radioembolization. A small phase pilot study found no benefit in combining Y-90 radioembolization with tremelimumab and durvalumab, being closed prematurely for futility [67].

Data regarding the combination of immunotherapy with other local or regional therapy options for MSS CRLM, such as hepatic arterial infusion pump, radiofrequency or microwave ablation and transarterial chemoembolization, are scarce. Presently, LM resection might be the preferred option where feasible, considering its potential for greater clinical benefits despite immunotherapy. Data from LiverMetSurvey, an international registry gathering information on patients undergoing surgical treatment for CRLM, present a 10-year survival rate of 25% for patients with resected LM [68].

A significant concern revolves around the optimal interval between surgery and commencing immunotherapy. An ongoing phase II multicenter study evaluating botensilimab as monotherapy and in combination with balstilimab versus standard treatments in participants with refractory metastatic CRC excluded patients with existing LM but allowed those with definitively treated LM (surgical resection, microwave ablation or radiofrequency or stereotactic body radiotherapy) if treated at least 6 months before enrolment [69]. While liver resections might suit a specific patient subset, they carry a high risk of recurrence. A shorter interval for initiating immunotherapy post-liver resection could enhance its efficacy against extrahepatic diseases like lung or lymph node metastases. This aligns with results from retrospective studies [36].

Emphasizing the judicious selection of patients for appropriate surgical treatments, including local therapies, one study evaluated incorporating magnetic resonance imaging (MRI) with gadoxetic acid alongside contrast computed tomography, resulting in a treatment plan change for 31% of 298 patients. These alterations mitigated both overtreatment and, importantly, undertreatment, likely improving long-term patient outcomes and generating cost savings. Nonetheless, concerns persist regarding the feasibility of routine preoperative MRI with gadoxetic acid evaluation in patients scheduled for local therapy of CRLM. Apart from training and protocol adjustments, delayed MRI acquisition could postpone surgical intervention, potentially allowing disease progression during the waiting period [70]. Therefore, a multidisciplinary team discussion involving highly proficient experts is imperative when planning treatment for MSS CRLM, especially when contemplating subsequent immunotherapy.

## Conclusion

Liver metastasis is a common occurrence in CRC, presenting a unique TME and imposing considerable therapeutic challenges. Numerous studies consistently show the resistance derived from CRLM to classical ICI, both alone and in combination with TKIs. As a result, the design of upcoming trials investigating immunotherapy in metastatic MSS CRC patients should consider incorporating liver metastasis as a stratification factor or contemplate excluding patients with active CRLM. In the pursuit of improved therapeutic outcomes, there is a growing interest in exploring new approaches beyond PDL1 and CTLA-4. This exploration is driven by the pressing need for effective treatment options for MSS patients affected by LM.

## Conflicts of interest

The authors declare no conflict of interest with the present manuscript.

## Funding

No funding was received for the present manuscript.

## Informed consent

No personal information of patients or relatives included this study.

## Author contributions

Conceptualisation, RDP; Methodology, MS and RDP; Investigation, MSA; MSD and RDP; writing original draft preparation, MSA; MSD and RDP; writing – review and editing, MSD and RDP; project administration, RDP; All authors have read and agreed to the published version of the manuscript.

## List of abbreviations

ANGPTL1, Angiopoietin-like protein 1; APC, Antigen-presenting cell; CAF, Cancer-associated fibroblast; CRC, Colorectal cancer; CRLM, Colorectal liver metastasis; DCR, Disease control rate; dMMR, Deficient mismatch repair; HSC, Hepatic stellate cell; ICI, Immune checkpoint inhibitor; KC, Kupffer cell; M1, Macrophage type 1; M2, Macrophage type 2; MRI, Magnetic resonance imaging; MSI, Microsatellite instable; MSS, Microsatellite stable; MDSC, Myeloid-derived suppressor cells; N1, Neutrophil type 1; N2, Neutrophil type 2; NK, Natural killer; ORR, Objective response ratio; PD1, Programmed death 1; PDL1, Programmed death-ligand 1; PFS, Progression free survival; TGF-beta, Transforming growing factor beta; TIL, Tumour infiltrating lymphocytes; TIM-3, T-cell immunoglobulin mucin 3; TKI, Tyrosine kinase inhibitor; TME, Tumour microenvironment; Treg, Regulatory T cell; VEGF, Vascular endothelial growing factor.

## Figures and Tables

**Table 1.** List of studies evaluating different strategies of immunotherapy in metastatic MSS CRC.

Author, Year	Strategy	Participants	Outcomes
**Immunotherapy alone**			
Brahmer *et al* (2010)	Phase I MDX-1106 (anti-PD1)	Various tumours 14/39 MSS CRC	1 CR, 2 PR
Overman *et al* (2016)	Phase II – CheckMate-142 Nivolumab + ipilimumab	28/83 MSS CRC	PFS 1.4 m
Chen *et al* (2020)	Phase II – CO.26 trial Durvalumab + tremelimumab	180, all MSS CRC	PFS 1.8 m
Garralda *et al* (2021)	Phase I Pembrolizumab + favezelimab	109, all MSS CRC	ORR 6.3% PFS 2.1 m
Bullock *et al* (2023)	Phase Ib Balstilimab + botensilimab	101, all MSS CRC	ORR 23% (without CRLM) versus 0% (with CRLM)
**Immunotherapy combined with other systemic therapies**			
Hellman *et al* (2019)	Phase Ib Atezolizumab + cobimetinib	Various tumours 84/152 MSS CRC	ORR 8%
Eng *et al* (2019)	Phase III – IMBlaze-370 Atezolizumab + comibetinib versus regorafenib	363, all MSS CRC	ORR 3% OS 8.8 versus 8.5 m *p* 0.99
Cousin *et al* (2020)	Phase II – REGOMUNE Avelumab + regorafenib	48, all MSS CRC	ORR 0% PFS 3.6 m
Gomez-Roca *et al* (2021)	Phase II – LEAP005 Pembrolizumab + lenvatinib	Various tumours 32/187 MSS CRC	ORR 22% PFS 2.3 m
Barzi *et al* (2022)	Phase I/II Pembrolizumab + regorafenib	73, all MSS CRC	ORR 0% PFS 2 m
Saeed *et al* (2023)	Phase I – CAMILLA Durvalumab + cabozantinib	Various tumours 17/35 MSS CRC	ORR 23.5% PFS 4.6 m
Fakih *et al* (2023)	Phase II – REGONIVO Nivolumab + regorafenib	70, all MSS CRC	ORR 7% PFS 1.8 m
Kawazoe *et al* (2023)	Phase III – LEAP017 Pembrolizumab + lenvatinib versus regorafenib or TFD/TPI	480, all MSS CRC	ORR 10.4% versus 1.7% OS 9.8 versus 9.2 m *p* 0.0379
**Immunotherapy combination with locoregional treatments**			
Wang *et al* (2020)	Pilot study Durvalumab + tremelimumab + radioembolization	9 all, MSS CRC	ORR 0% Closed for futility
Wang *et al* (2021)	Retrospective Immunotherapy ± CRLM resection	95 all, MSS CRC	PFS 3 versus 1.5 m
Parikh *et al* (2021)	Phase II Nivolumab + ipilimumab + radiotherapy	Various Tumours 40/65 MSS CRC	ORR 2.5%–10%

CR, Complete response; PR, Partial response; ORR, Objective response ratio; PFS, Progression free survival; OS, Overall survival; MSS, Microsatellite stable; CRC, Colorectal cancer, CRLM, Colorectal liver metastasis
